# High Heregulin Expression Is Associated with Activated HER3 and May Define an Actionable Biomarker in Patients with Squamous Cell Carcinomas of the Head and Neck

**DOI:** 10.1371/journal.pone.0056765

**Published:** 2013-02-28

**Authors:** David S. Shames, Juliet Carbon, Kim Walter, Adrian M. Jubb, Cleopatra Kozlowski, Tom Januario, Do An, Ling Fu, Yuanyuan Xiao, Rajiv Raja, Brittany Jiang, Ashi Malekafzali, Howard Stern, Jeff Settleman, Timothy R. Wilson, Garret M. Hampton, Robert L. Yauch, Andrea Pirzkall, Lukas C. Amler

**Affiliations:** 1 Oncology Biomarker Development, Genentech Inc., South San Francisco, California, United States of America; 2 Research Pathology, Genentech Inc., South San Francisco, California, United States of America; 3 Early Clinical Development, Genentech Inc., South San Francisco, California, United States of America; 4 Biostatistics, Genentech Inc., South San Francisco, California, United States of America; 5 Safety Assessment Pathology, Genentech Inc., South San Francisco, California, United States of America; 6 Molecular Diagnostics and Cancer Cell Biology, Genentech Inc., South San Francisco, California, United States of America; Virginia Commonwealth University, United States of America

## Abstract

**Purpose:**

Tumors with oncogenic dependencies on the HER family of receptor tyrosine kinases (RTKs) often respond well to targeted inhibition. Our previous work suggested that many cell lines derived from squamous cell carcinomas of the head and neck (SCCHNs) depend on autocrine signaling driven by HER2/3 dimerization and high-level co-expression of HRG. Additionally, results from a Phase I trial of MEHD7495A, a dual-action antibody that blocks ligand binding to EGFR and HER3, suggest that high-level *HRG* expression was associated with clinical response in SCCHN patients. Here we explore the hypothesis that high-level *HRG* expression defines a subpopulation of SCCHNs with activated HER3.

**Experimental Design:**

qRT-PCR expression profiling was performed on >750 tumors of diverse origin, including >150 therapy-naïve, primary, and recurrent SCCHNs. Activated HER3, defined by immunoprecipitation of phospho-HER3, was compared to *HRG* expression in SCCHN samples. Paracrine versus autocrine expression was evaluated using RNA-in situ hybridization.

**Results:**

SCCHN tumors express the highest levels of *HRG* compared to a diverse collection of other tumor types. We show that high *HRG* expression is associated with activated HER3, whereas low *HRG* expression is associated with low HER3 activation in SCCHN tumors. Furthermore, *HRG* expression is higher in recurrent SCCHN compared to patient-matched therapy naïve specimens.

**Conclusions:**

*HRG* expression levels define a biologically distinct subset of SCCHN patients. We propose that high-level expression of *HRG* is associated with constitutive activation of HER3 in SCCHN and thus defines an actionable biomarker for interventions targeting HER3.

## Introduction

Approximately 52,140 new cases of squamous cell carcinoma of the head and neck (SCCHN) were diagnosed and 11,460 people are estimated to have died from this disease in the United States last year [1]. Curative interventions for SCCHN include surgery, radiation, and combined radio-chemotherapy. The overall 5-year relative survival rate for primary SCCHN is approximately 60%. However, the 5-year relative survival rate is only 35% for patients diagnosed with metastatic disease [2]. The poor outcomes in patients with advanced SCCHN clearly indicate the need for more effective therapies in this population [3].

Signaling through the epidermal growth factor receptor (EGFR) pathway is a major driver of SCCHN [4]. EGFR is overexpressed in up to 90% of all SCCHN [5], [6]. EGFR inhibition with cetuximab has proven to be a successful therapeutic strategy, albeit with somewhat limited long-term clinical benefits due to intrinsic or acquired resistance [7]. Tyrosine kinase inhibitors (TKIs), such as erlotinib, gefitinib, and lapatinib, that target EGFR and or HER2 have been investigated in clinical studies of SCCHN but have not demonstrated a survival advantage in randomized trials [8], [9], [10].

Recently we showed that HER3 signaling also plays an important role in SCCHN [11], [12]. These preclinical studies suggested that constitutive, high-level activation of HER3 signaling can occur in the absence of direct genetic activation of *HER3* (e.g. mutation or amplification) or its ligand (mutation or amplification of Heregulin (*HRG*)). In addition, this activation did not occur as a result of mutation or amplification of the HER3 co-receptors EGFR or HER2. Rather, we demonstrated that chronic HER3 signaling was driven by high-level and potentially autocrine expression of the alpha and beta forms of Neuregulin 1 (NRG1), collectively known as (HRG) [13], [14] in cell lines derived from SCCHN. Moreover, in a recent phase I trial of an antibody that blocks ligand binding to EGFR and HER3, the two patients who exhibited partial responses had SCCHN tumors that expressed high levels of *HRG*, suggesting a potential causal relationship between high *HRG* expression and activity of MEHD7495A [12], [15]. Thus high expression of *HRG* could define a population of tumors that may have an oncogenic dependency on ligand-activated signaling via HER3. To further explore the hypothesis that high-level *HRG* expression defines a sub-population of SCCHN that may be sensitive to agents targeting HER3 and to identify other potential target indications, we evaluated the expression of *HRG* in large cohorts of patient samples from multiple solid tumor indications and surveyed the activation status of HER3 in SCCHN tumor samples.

## Materials and Methods

### Ethics Statement

#### First-line NSCLC samples

All patient samples were obtained as part of a completed Phase II clinical trial and is listed at http://clinicaltrials.gov/ct2/show/NCT00480831. All samples were collected and analyzed with written informed consent. The protocol for this trial was reviewed by an external review board (The Copernicus Group IRB; http://www.cgirb.com/) prior submission to the regulatory agencies as well as by the review boards associated with each site.

#### Second/third line NSCLC samples

All patient samples were obtained as part of a completed Phase II clinical trial and is listed at http://clinicaltrials.gov/ct2/show/NCT00854308. All samples were collected and analyzed with written informed consent. The protocol for this trial was reviewed by an external review board (The Copernicus Group IRB; http://www.cgirb.com/) prior submission to the regulatory agencies as well as by the review boards associated with each site.

#### First-line CRC samples

All patient samples were obtained as part of a completed Phase II clinical trial and is listed at http://clinicaltrial.gov/ct2/show/NCT00636610. All samples were collected and analyzed with written informed consent. The protocol for this trial was reviewed by an external review board (The Copernicus Group IRB; http://www.cgirb.com/) prior submission to the regulatory agencies as well as by the review boards associated with each site.

### Tissue Specimens

A total of 754 tumor specimens were used in this study: 127 SCCHN (All stages, primary and recurrent), 117 surgically resected NSCLC (Stage I–IV), 102 NSCLCs from patients with untreated metastatic disease (Stage III–IV), 82 NSCLCs from patients who went on to fail front-line standard-of-care but who ultimately received 2L therapy (Stage III–IV), 29 metastatic platinum refractory ovarian cancers, 149 therapy-naïve metastatic colorectal cancers, 44 primary and metastatic melanomas, and 29 samples from patients with triple-negative breast cancer (stage I–IV). A separate cohort of 19 fresh frozen SCCHNs were used to correlate phosphor-HER3 levels by IP-western with *HRG* transcript. In addition, a separate series of matched samples from a cohort of 28 patients (biopsies from initial diagnosis and at time of first recurrence) with recurrent SCCHN was obtained. Tables summarizing the available pathologic and demographic variables of these patients and their tumors may be found in the supplementary information. All samples except 1^st^ Line NSCLC, 2/3L NSCLC, and 1^st^ Line metastatic CRC (referenced under the Ethics Statement) were obtained from commercial sources following IRB approval (http://www.libertyirb.com/) and appropriate confirmation of written informed consent.

### Cell Lines and HRG Induction

Cell lines were obtained from commercial sources. All cell lines were genotyped and tested for mycoplasma prior to experimentation. Cell lines were incubated in RPMI1640 (Sigma) with 10% FBS (Hyclone) at 37 degrees Celsius in a humidified atmosphere with 5% CO_2_. For the HRG induction experiments, cells were plated in 10 cm dishes at 50% confluence and allowed to adhere to the plates overnight. Cells were then washed with serum-free medium and then incubated for 24 hours in RPMI1640 supplemented with 0.5% serum. The samples were treated with 10 µg/ml MEHD7495A or vehicle at 22 hours and then treated with 3 nm of HRG (Genentech protein synthesis core facility). After 10 minutes, the cells were washed and then lysed as described below. Western analysis was performed using standard protocols. The following antibodies were purchased from Cell Signaling Technologies: pHER3 (Y1289; #4791), AKT (#9272), pAKT (Ser473; #9271), pERK (T202/Y204; #9101), ERK (#9102). The total HER3 (sc-285) and HSP90 (sc-7947) antibodies were purchased from Santa Cruz Biotechnologies.

### Immunoprecipitations and Immunoblotting

For immunoprecipitation of tumor tissue, tumor content was verified by H&E requiring a minimum of 50% tumor (most samples had >75% tumor tissue). Tumors were minced on dry ice, then homogenized in chilled lysis buffer (BioWorld, 22040045-2) supplemented with phosphatase and protease inhibitors (Sigma, P5726-5ML, Roche, 13146100). 25 µl anti-HER3 antibody (Santa Cruz, sc-285-G) and 15 µl Dynabeads (Invitrogen, 100.07D) were added to 1–2 mg soluble protein per immunoprecipitation. Samples were then allowed to rotate overnight at 4°C. Beads were then separated using a magnet, and washed three times in lysis buffer. Protein was eluted in sample buffer (Invitrogen, NP0007 and NP0009) by boiling at 95°C for 5 minutes. Western blotting was performed using standard protocols. 15 µl of eluted protein was loaded per sample and immunodetection was performed using pHER3 (Y1289) (Cell Signaling Technology; #4791) or pan p-Tyr (#9441, Cell Signaling Technology). Total HER3 was evaluated in the tumor lysates prior to immunoprecipitation with sc-285-G.

### Tissue Processing for Nucleic Acids

For RNA extraction tissue sections were submerged in 300 µl RLT buffer (Qiagen) and homogenized using a gentleMACS Octo Dissociator (Miltenyi Biotec). The samples were then split in half for DNA prep (DNAeasy, Qiagen) and RNA prep (TriZol, Invitrogen), as per the manufacturer’s instructions.

### Fluidigm Expression Analysis

Gene expression analysis was performed on the cell lines and formalin-fixed paraffin embedded tumor samples using the BioMark 96×96 gene expression platform (Fluidigm). For the tumors, 2 µl of total RNA was reverse-transcribed to cDNA and pre-amplified in a single reaction using Superscript III/Platinum Taq (Invitrogen) and Pre-amplification reaction mix (Invitrogen). The pre-amplification reaction was performed at a final dilution of 0.05x original Taqman assay concentration (Applied Biosystems). The thermocycling conditions were as follows: 1 cycle of 50°C for 15 min, 1 cycle of 70°C for 2 min, then 14 cycles of 95°C for 15 sec and 60°C for 4 min.

Pre-amplified cDNA was diluted 1.94-fold and then amplified using Taqman Universal PCR MasterMix (Applied Biosystems) on the BioMark BMK-M-96.96 platform (Fluidigm) according to the manufacturer’s instructions. All samples were assayed in triplicate. Two custom-designed assays targeting the reference genes, AL-1377271 and VPS-33B, were included in the expression panel. Primer/probe sequences are available upon request. A mean of the Ct values for the two reference genes was calculated for each sample, and expression levels of *HRG* and *HER3* were determined using the delta Ct (dCt) method as follows: Mean Ct (Target Gene) - Mean Ct (Reference Genes).

### Analysis of Distribution of HRG Expression and Cut-off Determination

The bimodal distribution of log_10_ (*HRG*) in HNSCC motivates the fitting of the mixture of two normal distributions, one corresponding to the lack of over-expression and the other to over-expression. Let x_i_ denote the log_10_ *HRG* expression for the *i*th sample (*i* = 1,…,n). The likelihood for the mixture model is:
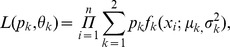
where 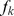 is the normal density function of the *k^th^* component, and (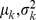) denote the corresponding mean and variance parameters. Maximum likelihood estimates of model parameters were obtained via the EM algorithm [16]. Briefly, the E-step computes the conditional probability that the *i^th^* sample belongs to the *k^th^* component of the mixture given the current parameter estimates. The M-step computes the mixing proportions, means and variances given the current probabilities. The process is then iterated to converge. Posterior probability of component membership was computed for each sample, and the cutoff was selected at the value where the posterior probabilities for the two components were equal. Model fitting was performed using the R package mixtools [17].

### Dual Color Chromogenic RNA-ISH

Dual color RNA in situ hybridization was performed by Advanced Cell Diagnostics (Fremont, CA). The *HRG* probe set has 31 pairs of oligos (62 total) covering nt 1082–3001 of transcript NM_013964. The *HER3* probe set is essentially a pool of two probe sets which together cover all of the transcript variants: 20 pairs of oligos (40 total) covering nt 1962–2945 of NM_001982. 14 pairs of oligos (28 total) covering nt 108–899 of NM_001005915. Probes designed against cyclophilin B (PPIB; positive) and the bacterial gene dihydrodipicolinate reductase (DapB; negative) were used as controls. Images were scanned by a Hamamatsu Nanozoomer Digital Slide scanner, running Nanozoomer software, with a 40× objective and 8-bit camera [2], [15]. The MatLab (MathWorks, Natick, MA) scoring algorithm consists of the following steps: A region of interest with a minimum of 75% tumor cells was manually defined by a pathologist for each section, then a haematoxylin mask was created to identify nuclei, followed by a blue mask (*HRG*) and a red mask (*HER3*) was applied. Individual “cells” were defined by the haematoxylin mask in order to unambiguously separate cells whereupon blue or red dot counts were tabulated for each cell.

Scanned images were also analyzed using Definiens Developer (Munich, AG), using the RGB (red, green and blue) spectra. The same region of interest was used for analysis as in the MATLAB method. The region was subdivided into tiled regions of approximately 300 um height and width and analyzed at full resolution. Both color intensity (balance of red, green, and blue intensity values) and object size were used as criteria to distinguish between cell nuclei, *HRG*, and *HER3*, from background. Heavily overlapping nuclei, which were impossible to spectrally separate were excluded from analysis, in order to avoid bias.

Both methods were used to derive each of the following groups of cells*: HER3*+/*HRG*+, *HER3*+/*HRG*−, *HER3*−/*HRG*+, and *HER3*−/*HRG*−. Autocrine signal was defined as (*HER3*+/*HRG*+)/((*HER3*+/*HRG*+)+(*HER3*+/*HRG*−)+(*HER3*−/*HRG*+)).

## Results

To identify potential target indications and patient populations that might be sensitive to HER3 inhibition, we profiled the expression of *HRG* and *HER3* in more than 750 individual tumor specimens using a high throughput multiplex qRTPCR system [18]. These tumor specimens included a large cohort of both primary and metastatic squamous and non-squamous NSCLCs derived from patients eligible for first and second line chemotherapy. In addition, we analyzed samples from patients with metastatic colorectal carcinoma (CRC), therapy-naïve and recurrent SCCHN, surgically resected melanoma, surgically resected triple-negative breast (TNB) cancer, and surgically resected ovarian cancers including those from patients with primary and acquired platinum-refractory disease (Pl/R Ova) (Table S1).

**Figure 1:**
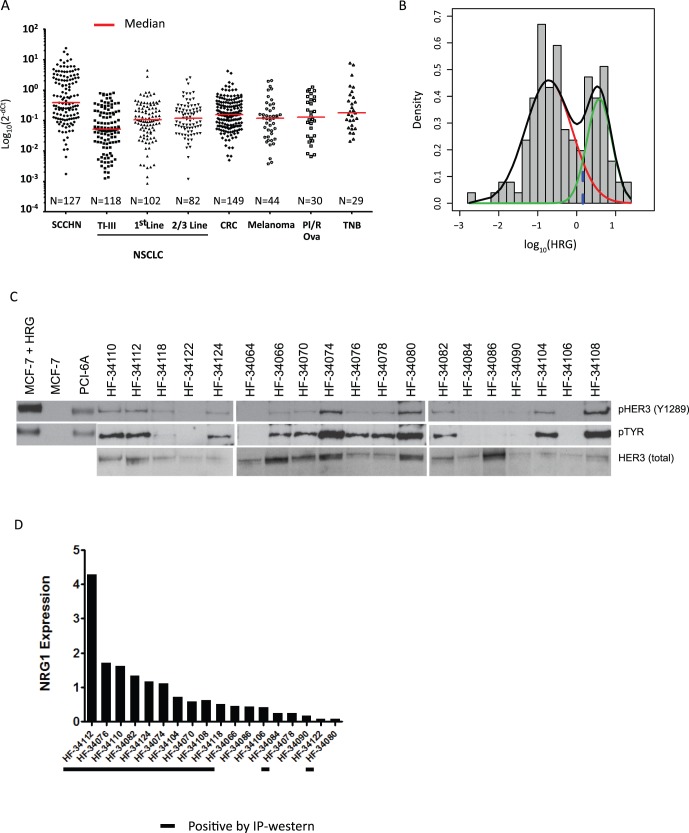
Analysis of *HRG* expression in the indicated epithelial tumors. **A)** qRTPCR of *HRG* in the indicated tumor types shows that SCCHNs have the highest median expression of HRG. NSCLC = non-small cell lung cancer; TI–III = pathological stage I–III; CRC = metastatic colorectal carcinoma; Pl/R Ova = platinum refractory ovarian cancers; SCCHN = squamous cell carcinoma of the head and neck; TNB = triple negative breast cancer. B) Statistical analysis of HRG expression in SCCHNs. The two mixture distributions and their joined distribution are depicted in red, green and black lines above. The dotted line indicates the inflection point between the high and low components of expression of HRG in SCCHN, which is set at 0.3689 on the logarithm scale. The sensitivity of identifying HRG overexpressed population using a cutoff of 0.3689 in the logarithm scale (∼1.45 on the linear scale) is 90.8% and the attendant specificity is 93.4%. C) IP-westerns for pHER3 (Y1289), pTyr, and total HER3 in 19 fresh frozen SCCHNs. MCF7 is a negative control; MCF7+ HRG and PCI6A are positive controls. The controls for the pTyr blot were run separately whereas the controls for the pHER3 were run concurrently. D) qRTPCR for HRG in 18/19 SCCHNs (overlap with the IP-western). Black lines below the x-axis indicate the tumors with detectable pHER3 by IP-western.

SCCHNs expressed the highest median levels of *HRG* compared to all other tumor types examined (Figure 1A; Figure S1A). In addition, a significant subset (approx. 40%) of these SCCHNs expressed higher levels of *HRG* than any other tumor type (Mann-Whitney test p<0.0001; Figure S1A). Furthermore, *HRG* expression exhibited a bimodal distribution in SCCHN when plotted on a log_10_ scale (Figure 1B, Figure S1A). A two-component Gaussian mixture distribution was used to estimate the inflection point between high and low expression, which was found to be near 1.5 on arbitrary linear scale. The misclassification rates, based on the fitted models, were 9.2% and 6.6% for the high expression and low expression populations, respectively.

We have previously demonstrated that in some SCCHN cell lines high-level co-expression of HRG and HER3 leads to constitutive HER3 signaling, which can be blocked by the HER3 arm of MEHD7495A [11]. We have also shown that MEHD7495A blocks ligand dependent activation of both HER3 and EGFR *in vivo* [12]. To confirm and extend our previous findings, we serum-starved several cell lines for 24 hours and then induced them using HRG with or without pre-incubation with MEHD7495A. As expected, HRG induced pHER3 as well as downstream activation of AKT in most cell lines (Figure S1C). MEHD7495A blocked this activation in all lines except EBC-1, which is known to harbor an amplification of *c-MET*.

To determine if high *HRG* expression was associated with activated HER3 in SCCHN tumors, we performed qRT-PCR for *HRG* and immunoprecipitation (IP) of total HER3 followed by Western blot for pHER3 and p-Tyrosine in fresh frozen tumor specimens from patients with therapy naïve SCHNN (Figure 1C & D; Table S1). We selected pHER3 as a read-out for this association because it is the most proximal and experimentally feasible (in human tumors) measure of ligand-activation of HER3 and thus the most relevant endpoint to the mechanism of action for MEHD7495A. All tumors with high *HRG* expression (at or near the low-point within the bimodal distribution) were positive for pHER3 by IP-Western; conversely, 5/7 tumors with the lowest expression of *HRG* were negative for pHER3 (two-tailed sign test, P = 0.0386).

**Figure 2:**
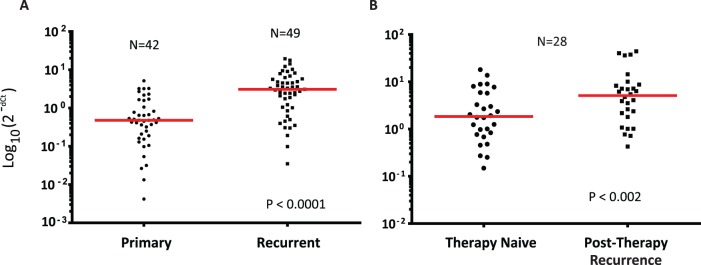
Expression of *HRG* in primary vs. recurrent SCCHN. A) qRTPCR analysis comparing the levels of *HRG* expression in unmatched primary and recurrent SCCHN specimens. B) qRTPCR analysis comparing the levels of *HRG* expression in matched primary and recurrent SCCHN. In both cases *HRG* levels appear to be higher in the recurrent disease setting compared to primary resected SCCHN.

To further characterize SCCHN patient populations with tumors that show high *HRG* expression we obtained tumor samples from patients with surgically resectable SCCHN as well as tumor samples from patients with recurrent disease. *HRG* expression was higher in the recurrent setting compared to primary resectable disease (Figure 2A). These findings could not be explained by differences in site of origin, HPV status, or any other available clinicopathological information (data not shown).

The finding that *HRG* expression may differ in primary versus recurrent disease could suggest that *HRG* expression increases as a consequence of prior therapy, or because high *HRG* expression is a prognostic factor associated with an increased likelihood of recurrence in patients with SCCHN. To explore this question further, we obtained a series of patient matched primary tumors and relapse specimens and compared *HRG* expression in both cohorts by qRTPCR. As shown in Figure 2B (Table 1 & 2), *HRG* expression is significantly higher in the recurrent setting compared to the primary disease in matched patient specimens (Wilcoxon signed rank test; P = 0.002).

**Table 1:** Detailed evaluation of *HRG* and *HER3* expression in matched therapy naïve and post-chemotherapy SCCHNs.

Primary•	NRG1	HER3	% Autocrine			Recurrance†	NRG1	HER3	% Autocrine		
			MatLab	Tumor Cells	Tumor Area				MatLab	Tumor Cells	Tumor Area
RxN 1	0.77	2.06	8.43%	38.0%	19.2%	PC 1	2.38	5.70	10.1%	36.8%	27.5%
RxN 2	13.71	3.36	4.41%	21.3%	10.3%	PC 2	4.09	5.83	4.3%	17.9%	8.3%
RxN 3	1.77	4.88	7.62%	27.6%	13.6%	PC 3	7.18	6.15	15.0%	36.9%	24.8%
RxN 4	8.99	0.62	#N/A	#N/A	#N/A	PC 4	36.13	2.02	10.0%	34.6%	24.0%
RxN 5	2.03	11.68	#N/A	#N/A	#N/A	PC 5	3.53	12.44	12.7%	40.4%	25.0%
RxN 6	0.46	6.58	3.21%	41.9%	29.7%	PC 6	0.77	6.89	2.1%	42.9%	27.9%
RxN 7	3.28	3.07	#N/A	#N/A	#N/A	PC 7	6.32	5.49	5.5%	30.0%	15.7%
RxN 8	1.92	4.23	23.96%	47.6%	39.1%	PC 8	3.90	5.50	#N/A	#N/A	#N/A
RxN 9	1.74	1.46	17.36%	40.3%	31.5%	PC 9	40.27	6.63	6.9%	37.5%	24.7%
RxN 10	1.01	3.09	5.51%	35.7%	23.3%	PC 10	1.80	3.95	#N/A	#N/A	#N/A
RxN 11	5.91	5.10	7.97%	31.8%	20.9%	PC 11	6.93	9.09	2.9%	23.9%	12.7%
RxN 12	7.71	4.35	9.86%	31.3%	18.9%	PC 12	7.14	7.66	1.8%	10.9%	3.5%
RxN 13	0.84	6.16	14.92%	54.9%	45.9%	PC 13	5.44	5.69	14.0%	32.3%	18.7%
RxN 14	1.25	0.34	5.36%	26.7%	17.2%	PC 14	2.17	2.40	#N/A	#N/A	#N/A
RxN 15	8.88	2.14	0.29%	7.8%	3.7%	PC 15	14.43	3.13	6.6%	26.5%	14.5%
RxN 16	18.10	7.72	27.30%	58.6%	38.5%	PC 16	6.19	2.91	1.9%	13.0%	5.9%
RxN 17	0.98	7.12	16.57%	46.0%	37.6%	PC 17	1.08	8.24	11.0%	37.1%	27.1%
RxN 18	0.25	1.33	9.63%	53.9%	39.3%	PC 18	0.72	4.41	8.0%	67.1%	56.5%
RxN 19	0.48	2.53	#N/A	#N/A	#N/A	PC 19	1.02	2.81	17.8%	62.4%	48.5%
RxN 20	5.75	3.90	#N/A	#N/A	#N/A	PC 20	44.21	3.28	14.7%	51.3%	43.5%
RxN 21	0.15	2.32	7.75%	35.6%	22.7%	PC 21	4.76	5.35	13.9%	47.3%	37.0%
RxN 22	0.68	0.03	1.45%	8.9%	3.0%	PC 22	0.43	0.07	0.4%	16.5%	6.4%
RxN 23	2.68	2.16	18.50%	60.2%	48.3%	PC 23	1.01	8.17	4.5%	43.0%	30.0%
RxN 24	3.01	1.34	1.40%	41.0%	28.2%	PC 24	8.25	2.03	22.1%	56.6%	44.0%
RxN 25	0.27	2.40	0.30%	29.9%	21.1%	PC 25	2.62	1.90	#N/A	#N/A	#N/A
RxN 26	2.35	11.53	18.58%	57.9%	49.8%	PC 26	37.51	2.39	4.4%	31.5%	20.9%
RxN 27	8.01	7.47	25.61%	51.2%	39.7%	PC 27	10.06	5.41	48.3%	71.3%	61.7%
RxN 28	1.24	4.95	8.93%	26.9%	16.9%	PC 28	8.72	1.71	11.3%	30.0%	21.1%

• RxN corresponds to therapy naïve patients;

† PC corresponds to Post-Chemo patients.

% Autocrine cells were based on tumor cell content only; qRTPCR was performed on macrodissected specimens as outlined in the methods section.

**Table 2:** Summary of the Relationship Between *HRG* Expression and the Extent of Autocrine versus Paracrine Expression in Matched Therapy Naïve Primary Specimens and Post Therapy Biopsies Taken at Recurrence.

Comparison	Rx Naïve	Post-Rx	Significance*
HRG	3.72 (1.99–4.45)	9.61 (4.6–14.62)	0.002
HER3	4.07 (2.9–4.24)	4.9 (3.84–5.96)	0.026
Autocrine (MatLab)	10.65% (7.12–14.19)	10.43% (6.62–14.59)	NS
Autocrine (Definiens Cells)	38.04% (31.68–44.41)	37.4% (30.59–44.21)	NS
Autocrine (Definiens Area)	26.89% (21.01–32.77)	26.25% (19.65–32.85)	NS

* Wilcoxon rank sign test for paired samples.

Preclinical data from a recent report suggested that identifying tumors with autocrine biology may be important in predicting response to agents targeting HER family receptors [11]. To evaluate this hypothesis, we first examined the performance of a dual-color assay RNA *in situ* hybridization (ISH) assay in cell lines with previously defined expression levels of *HER3* and *HRG* (Figure S2). Overall there was strong agreement between qRTPCR and ISH for the levels of both *HER3* and *HRG.* Consistent with previously published data, the MCF7 cell line showed abundant levels of *HER3* and no detectable *HRG,* whereas both PCI-6A and CHL-1 showed high levels of both *HER3* and *HRG* [11], [12]. In addition, both PCI-6A and CHL-1 had significant numbers of autocrine cells, whereas H358 was predominantly paracrine (Figure S2).

**Figure 3:**
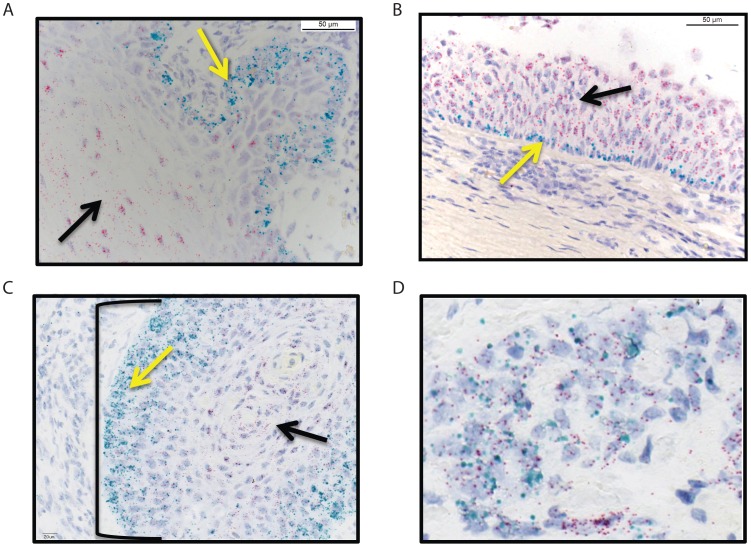
Dual color *in situ* hybridization for *HER3* and *HRG* in benign and malignant tissue shows that *HER3* and *HRG* are typically expressed by different cells. (HER3 = red; HRG = cyan) A) Normal squamous epithelium; B) Normal upper respiratory epithelium; C) well differentiated SCCHN; D) autocrine expression of *HRG* and *HER3*. In panels A & B, the yellow arrows point to basal cells and the black arrows point to squamous epithelium or more differentiated cells within the respiratory tract. In panel C, the bracketed structure is a squamous morule, the yellow arrow again points to “basal-like” tumor cells whereas the black arrow identifies spiny cells. Panel D represents anaplastic autocrine cells, characteristic of a poorly differentiated SCCHN. Magnification is 200x for A–C and 400× for part D.

To determine the extent of autocrine versus paracrine expression of *HER3* and *HRG* expression in SCCHNs we employed a similar (same probes with chromogenic versus fluorescent probes) dual-color chromogenic RNA *in situ* hybridization assay designed to evaluate *HER3* and *HRG* transcript location and abundance in clinically relevant FFPE tissues. Qualitative examination of benign squamous epithelium suggested that expression of *HRG* (cyan) was limited to the basal layer, whereas *HER3* (red) expression was only seen focally in normal stratified squamous epithelium (Figure 3A). In contrast, in the spiny layer, only *HER3* expression was observed. A similar pattern of expression was seen in the pseudostratified upper respiratory tract epithelium where *HRG* expression was limited to the basal layer and *HER3* expression was restricted to the upper layers of the epithelium (Figure 3B; Figure S3A & B). At the single cell level in benign tissue, absent a source of *HRG*, most cells and tissues express relatively consistent levels of *HER3*, whereas in the presence of *HRG*, there is a gradient of *HER3* expression that increases the farther away the cells are from the source of *HRG*. More generally, there is an inverse relationship between the expression of *HER3* and *HRG* both at the level of individual cells as well as in the spatial orientation of cells that express either of these two transcripts.

We found clear evidence of paracrine expression of *HRG* and *HER3* in some well-differentiated head and neck squamous cell carcinomas, recapitulating the expression patterns seen in normal tissue (Figure 3C). In other more poorly differentiated squamous cell carcinomas the relationship between basal cell-specific expression of *HRG* and spiny cell-specific expression of *HER3* was lost, consistent with the more disordered architecture, with evidence for both autocrine and paracrine expression patterns (Figures S3E, F, & G). Finally, we identified cases where the majority of *HER3*/*HRG* expressing cells appeared to be autocrine (Figure 3D).

To determine if high *HRG* expression was specifically associated with either autocrine or paracrine expression, we compared qRTPCR with RNA-ISH for *HRG* and *HER3* in the same samples represented in Figure 2B. We employed three different quantitative imaging algorithms to identify autocrine cells (see methods for details). While each algorithm differed in the relative proportion of autocrine component for each tumor, the methods ranked the different tumors similarly (Spearman r: ∼0.75–0.96 for all pairwise combinations, p<0.0001 in all cases). This suggests that the algorithms were differentiating autocrine and paracrine cells in a similar way, albeit with potentially different sensitivities (Table 1).

**Figure 4:**
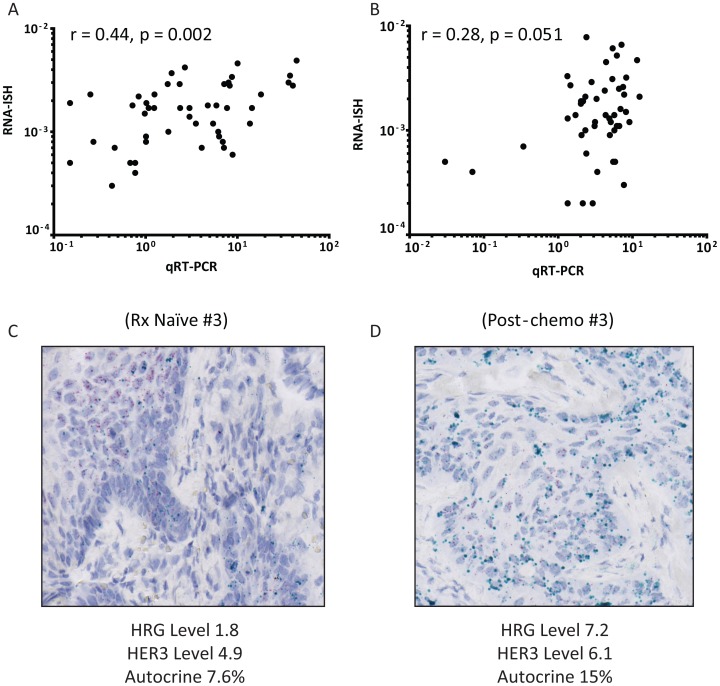
Quantitative comparison between RNA-ISH and QRTPCR. A & B) Pairwise analysis of RNA-ISH and qRTPCR of *HRG* and *HER3* in primary and recurrent SCHNNs. Spearman rank correlations and p-values are shown on the graphs. C) Representative RNA-ISH for a matched primary and recurrent section. The primary therapy naïve specimen (#3) was considered to *HRG* positive (2+) with frequent positive cells throughout squamous morules, but accentuated in basal cells. The tumor was also considered positive for *HER3* (2+) with frequent positive cells throughout squamous morules, but accentuated in differentiated cells. This tumor was considered predominantly paracrine, but harbored occasional cells that expressed both *HRG* and *HER3*. Post-chemo Specimen #3 was also considered positive for *HRG* (2+) with frequent positive cells throughout squamous morules, but accentuated in basal cells. This sample was also positive for *HER3* (2+) with frequent positive cells throughout squamous morules, but accentuated in differentiated cells. Her3+/Nrg1− and Her3+/Nrg1+ seen in equal measure. This finding of increased autocrine character was also evident using quantitative imaging (Table 1). (HER3 = red; HRG = cyan). Magnification is 400x.

Next we compared the RNA-ISH data to qRTPCR in tumor tissues. There was a strong, significant positive Spearman correlation between qRTPCR and RNA-ISH for *HRG* (r = 0.4; p = 0.002) and a lower, near significant, positive correlation between qRTPCR and RNA-ISH for *HER3* (ρ = 0.2; p = 0.051) (Figure 4A & B). In contrast to our findings in cell lines (Figure S2 & [11]) we did not observe a strong association between high-level *HRG* expression as determined by qRTPCR and the proportion of cells with autocrine expression of *HRG* and *HER3* as determined by RNA-ISH (Spearman r: 0.26 (p = 0.22), 0.03 (p = 0.91), respectively; Tables 1 & 2). In addition, while we did observe an increase in autocrine expression of *HER3* and *HRG* between matched therapy-naïve and recurrent SCCHNs in some cases (Figure 4C), unlike *HRG* expression (p<0.002; Figure 2B), this was not statistically significant overall (Table 2; methods).

## Discussion

In this report we evaluated the quantitative relationship between *HER3* and *HRG* expression in more than 700 hundred epithelial tumors of diverse tissue origin. We showed that a substantial subset of SCCHNs expressed significantly higher levels of *HRG* compared to all other tumor types examined. *HRG* expression has a unique bimodal distribution in SCCHN, with approximately 40% of SCCHN tumors expressing higher levels of *HRG* than all other tumor types. This pattern of expression is reminiscent of *HER2* expression in breast cancer, where *HER2* expression levels are at least an order of magnitude higher in HER2 positive breast cancers compared to other types of breast cancers. However, unlike HER2 in breast and gastric cancers, *HRG* overexpression does not appear to be a function of gene amplification [4], [19], [20].

We further showed that high *HRG* expression was associated with pHER3, possibly indicating active HER3 signaling in these particular SCCHN tumors. We found that pHER3 was detectable in all cases where *HRG* was expressed at or near the low point of the bimodal distribution in the SCCHN patients. In some cases pHER3 was detected in samples with *HRG* levels that were somewhat below the low point of the distribution. Interestingly, the low point of the bimodal distribution was similar in multiple independent data sets, potentially reflecting biologically distinct populations of SCCHN patients. Thus, based on the properties of the distribution of *HRG* expression and the biochemical association between *HRG* expression and pHER3 activity, it seems reasonable to hypothesize that a potential cut-off for identifying patients with SCCHN that might benefit from HER3-directed therapeutic intervention could be defined by comparing *HRG* expression levels with clinical activity in future clinical studies.

Importantly, two of the lowest *HRG* expressing tumors also had detectable pHER3. It has been shown that HRG-independent phosphorylation of HER3 can occur through heterodimerization with EGFR or other RTKs such as c-Met [1]. Patients with tumors exhibiting HRG-independent activation of HER3 are unlikely to benefit from HER3 directed therapies and thus it is important to note that using HRG as a predictive marker would effectively exclude this patient population [12].

Using a novel dual color ISH assay, we demonstrated that high-level expression of *HRG* occurs in the context of tumors with both paracrine and autocrine phenotypes, suggesting that a subset of SCCHNs may originate from cell lineages that are programmed to express abundant *HRG*. To our surprise this lineage appears to be the basal cells in squamous epithelium and not the stroma, whereas *HER3* is expressed almost exclusively by the more differentiated squamous epithelium. It will be of interest to determine if other tumor types derived from basal cells within squamous epithelium also express similar phenotypes. As a result of the heterogeneous nature of *HRG* expression in SCCHNs and the absence of clinical activity to inform this analysis, it is impossible to prospectively define whether a predominantly autocrine biology will be relevant to treatment with therapeutics that inhibit HRG driven HER3 signaling, but it is an important question that should be tested in clinical trials of such agents.

We showed that that *HRG* expression is higher in recurrent tumor specimens compared to matched and unmatched primary tumors. These findings, taken together with the data above, suggest that *HRG* expression may be both predictive of response to HER3 inhibitors and prognostic for recurrence of SCCHN. It will be important to consider the evident interaction between the prognostic and predictive properties of *HRG* expression in the design of future clinical trials.

In conclusion, we suggest that *HRG* expression levels define a statistically and biologically distinguishable subset of SCCHN patients. We propose that high-level expression of *HRG* is associated with constitutive activation of HER3 in SCCHN and thus defines an actionable biomarker for drugs that inhibit this important oncogene.

## Supporting Information

Figure S1 **Analysis of** ***HRG*** **Expression and the effect of MEHD7495A.** A) *HRG* expression depicted as a histogram demonstrates a bimodal distribution in HNSCC. Red bars indicate SCCHN; blue bars indicate all other cancer types examined including NSCLC, CRC, platinum refractory ovarian cancers, tripe negative breast cancers, and melanoma. The percentage of samples within a given in the overall distribution is indicated on the y-axis; transcript level (2^−dCt^) is indicated on the x-axis. B) Analysis of *HER3* expression in the indicated epithelial tumors. NSCLC = non-small cell lung cancer; TI–III = pathological stage I–III; CRC = metastatic colorectal carcinoma; Pl/R Ova = platinum refractory ovarian cancers; SCCHN = squamous cell carcinoma of the head and neck; TNB = triple negative breast cancer. C) MEHD7495A blocks HRG-dependent activation of HER3 signaling. Western blots of HER3, pHER3, AKT, pAKT, ERK, pERK, and HSP90 are shown. Treatment conditions are indicated above each lane. Cells were treated as described in the methods section.
(PPTX)

Figure S2 **Validation of dual-color** ***In Situ*** **hybridization assay for** ***HRG*** **and** ***HER3*** **in the indicated cell lines.** A) Fluorescent micrographs of MCF7 cells treated with probes corresponding to the *HER3* (*ERBB3*) and *HRG* (*NRG1*) transcript show that MCF7 does not express detectable *HRG* but has abundant expression of *HER3*; B) H358; C) PCI-6A; D) CHL-1.
(PPTX)

Figure S3 **Additional examples of the colormetric dual color** ***In Situ*** **hybridization assay for** ***HRG*** **and** ***HER3*** **in benign and malignant head and neck tissues.**
(TIF)

Table S1 **Select clinical and demographic information on patient samples evaluated for** ***HRG*** **and** ***HER3*** **expression.**
(XLSX)
