# Correction: High Heregulin Expression Is Associated with Activated HER3 and May Define an Actionable Biomarker in Patients with Squamous Cell Carcinomas of the Head and Neck

**DOI:** 10.1371/annotation/63f57c72-c869-4ef5-94d1-3cbd6c2e3678

**Published:** 2013-06-18

**Authors:** David S. Shames, Juliet Carbon, Kim Walter, Adrian M. Jubb, Cleopatra Kozlowski, Tom Januario, An Do, Ling Fu, Yuanyuan Xiao, Rajiv Raja, Brittany Jiang, Ashi Malekafzali, Howard Stern, Jeff Settleman, Timothy R. Wilson, Garret M. Hampton, Robert L. Yauch, Andrea Pirzkall, Lukas C. Amler

The name of the seventh author was spelled incorrectly. The correct name is: An Do. 

