# Supplementary material for: High Heregulin Expression Is Associated with Activated HER3 and May Define an Actionable Biomarker in Patients with Squamous Cell Carcinomas of the Head and Neck
Source: PLoS One. 2013 Feb 28;8(2):e56765. doi: 10.1371/journal.pone.0056765 (PMC3586092; doi:10.1371/journal.pone.0056765)
Supplement: Figure S1 — Analysis of HRG Expression and the effect of MEHD7495A. A) HRG expression depicted as a histogram demonstrates a bimodal distribution in HNSCC. Red bars indicate SCCHN; blue bars indicate all other cancer types examined including NSCLC, CRC, platinum refractory ovarian cancers, tripe negative breast cancers, and melanoma. The percentage of samples within a given in the overall distribution is indicated on the y-axis; transcript level (2−dCt) is indicated on the x-axis. B) Analysis of HER3 expression in the indicated epithelial tumors. NSCLC = non-small cell lung cancer; TI–III = pathological stage I–III; CRC = metastatic colorectal carcinoma; Pl/R Ova = platinum refractory ovarian cancers; SCCHN = squamous cell carcinoma of the head and neck; TNB = triple negative breast cancer. C) MEHD7495A blocks HRG-dependent activation of HER3 signaling. Western blots of HER3, pHER3, AKT, pAKT, ERK, pERK, and HSP90 are shown. Treatment conditions are indicated above each lane. Cells were treated as described in the methods section. (PPTX) [file pone.0056765.s001.pptx]

## Slide 1
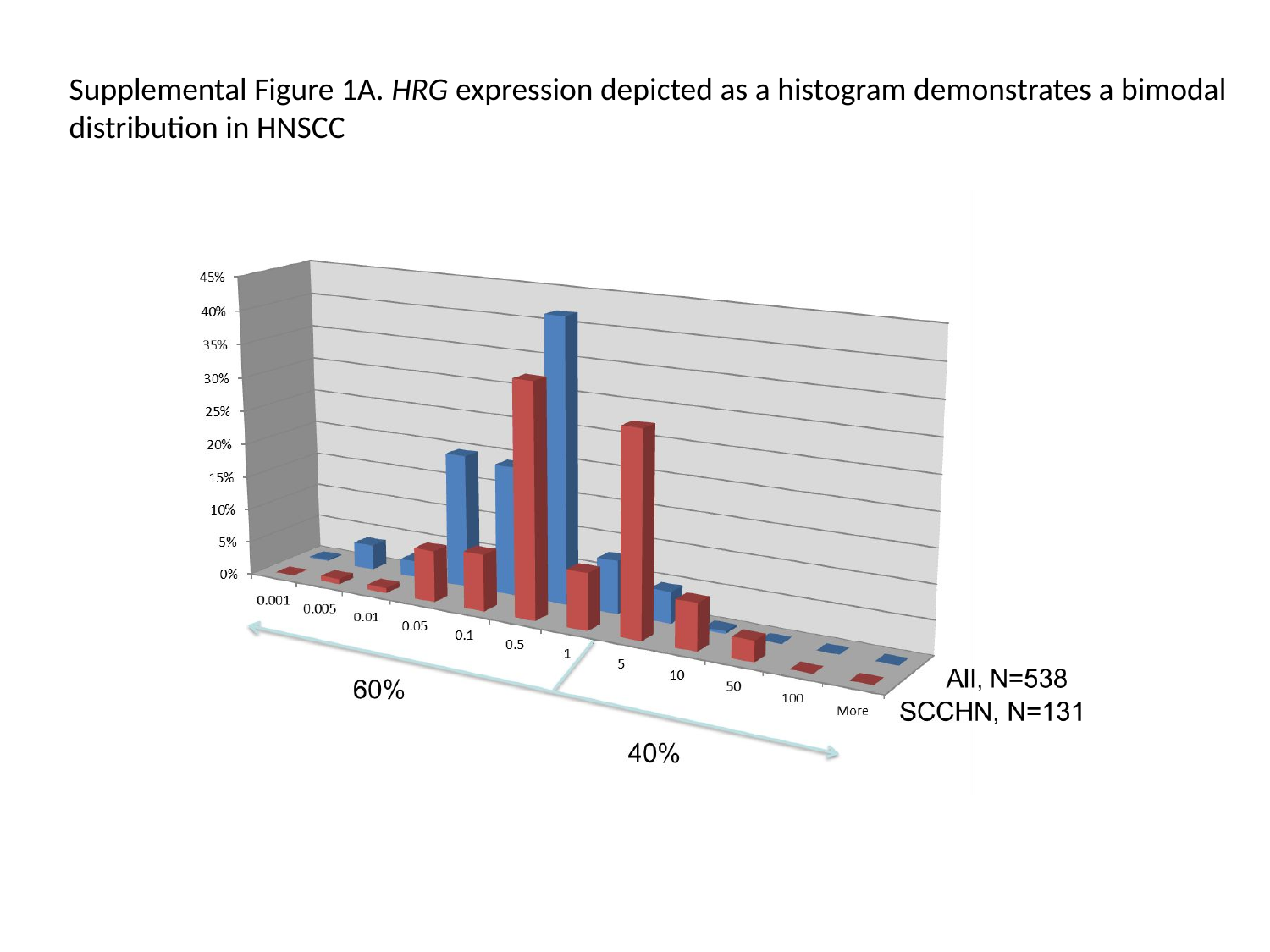

Supplemental Figure 1A. HRG expression depicted as a histogram demonstrates a bimodal distribution in HNSCC

## Slide 2
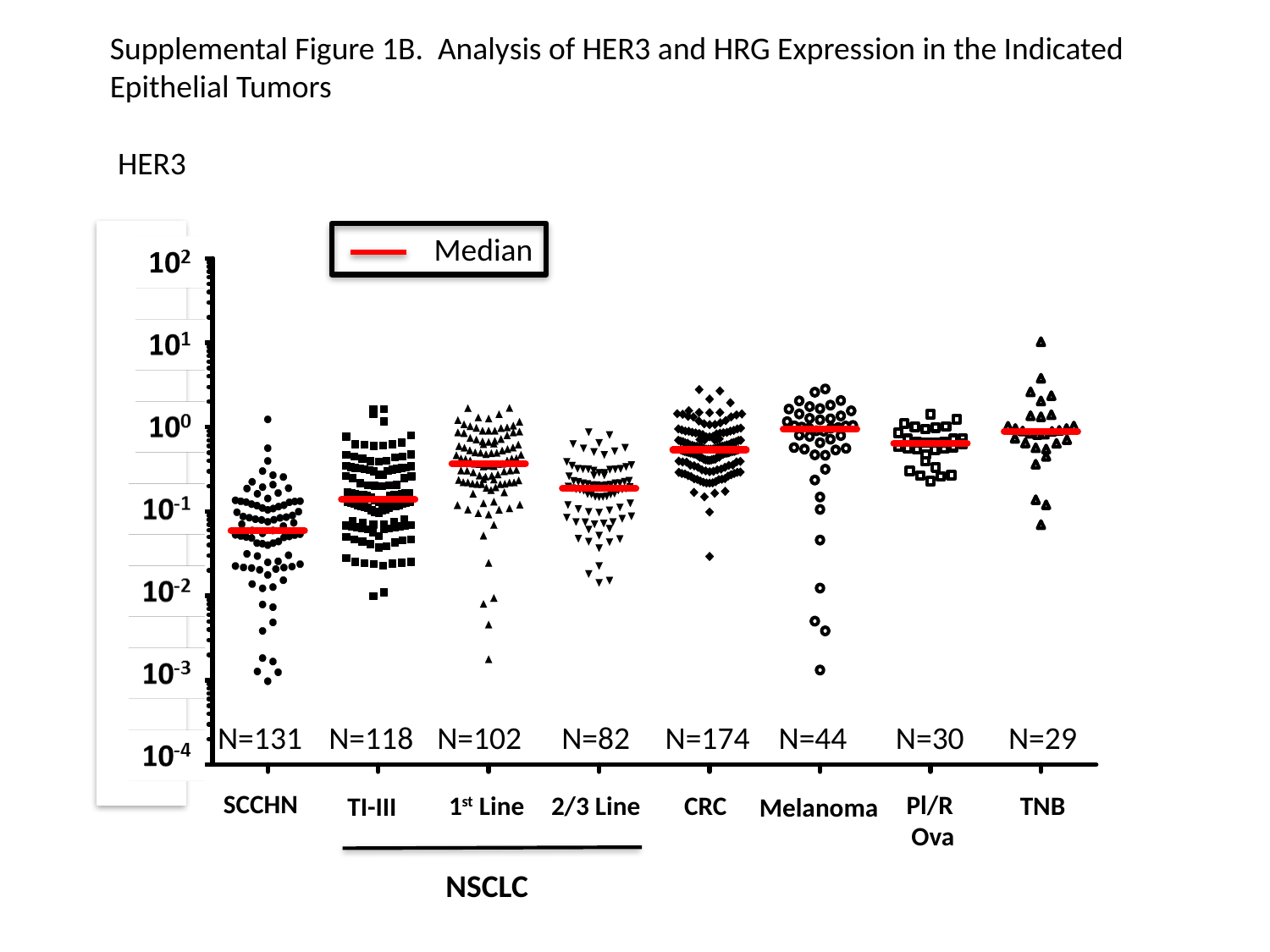

Supplemental Figure 1B. Analysis of HER3 and HRG Expression in the Indicated Epithelial Tumors
HER3
Median
N=131
N=118
N=102
N=82
N=174
N=44
N=30
N=29
Pl/R
Ova
Melanoma
SCCHN
CRC
TNB
1st Line
2/3 Line
TI-III
NSCLC

## Slide 3
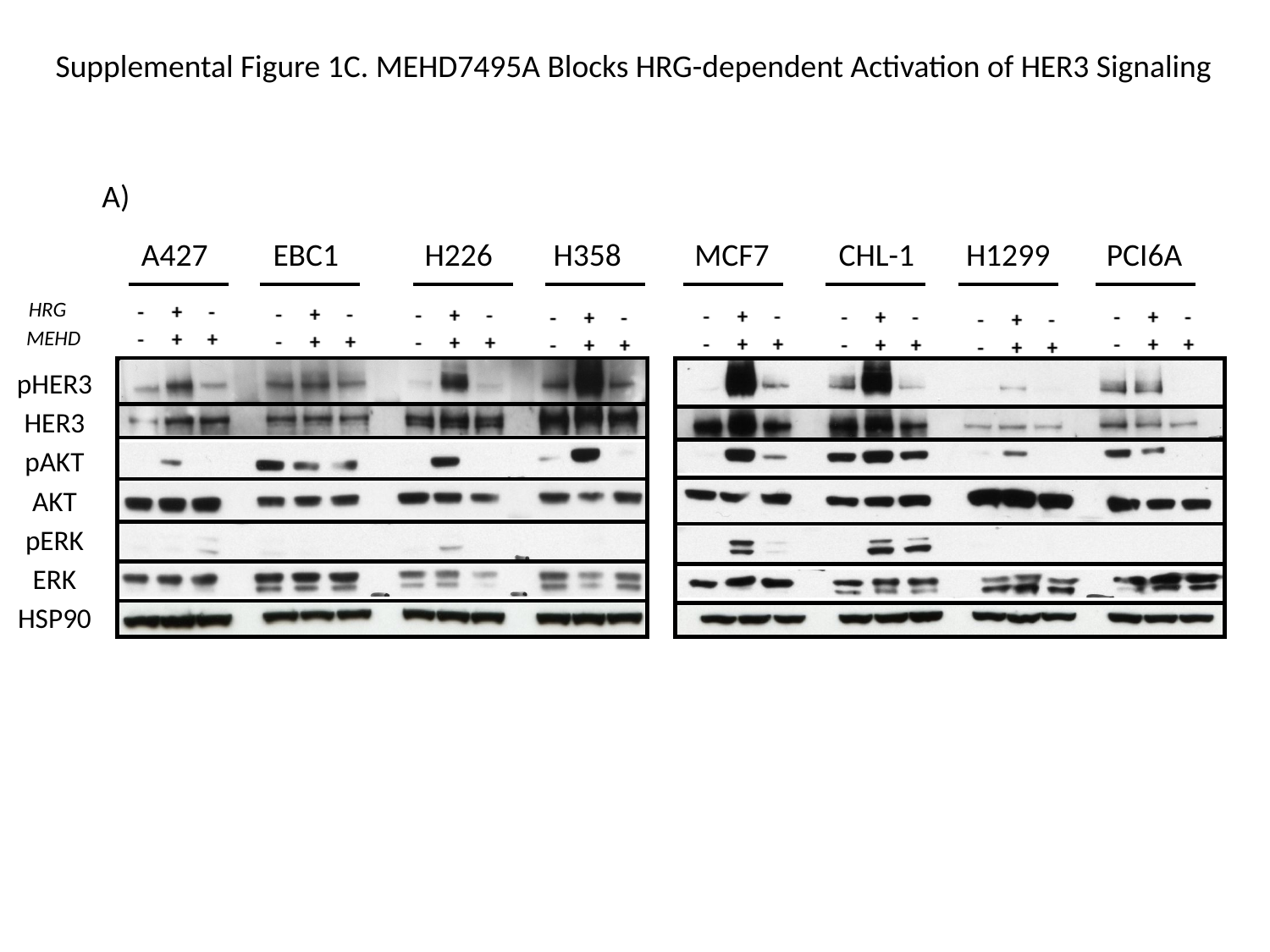

Supplemental Figure 1C. MEHD7495A Blocks HRG-dependent Activation of HER3 Signaling
A)
A427
EBC1
H226
H358
MCF7
CHL-1
H1299
PCI6A
HRG
MEHD
pHER3
HER3
pAKT
AKT
pERK
ERK
HSP90
