# Supplementary material for: High Heregulin Expression Is Associated with Activated HER3 and May Define an Actionable Biomarker in Patients with Squamous Cell Carcinomas of the Head and Neck
Source: PLoS One. 2013 Feb 28;8(2):e56765. doi: 10.1371/journal.pone.0056765 (PMC3586092; doi:10.1371/journal.pone.0056765)
Supplement: Figure S2 — Validation of dual-color In Situ hybridization assay for HRG and HER3 in the indicated cell lines. A) Fluorescent micrographs of MCF7 cells treated with probes corresponding to the HER3 (ERBB3) and HRG (NRG1) transcript show that MCF7 does not express detectable HRG but has abundant expression of HER3; B) H358; C) PCI-6A; D) CHL-1. (PPTX) [file pone.0056765.s002.pptx]

## Slide 1
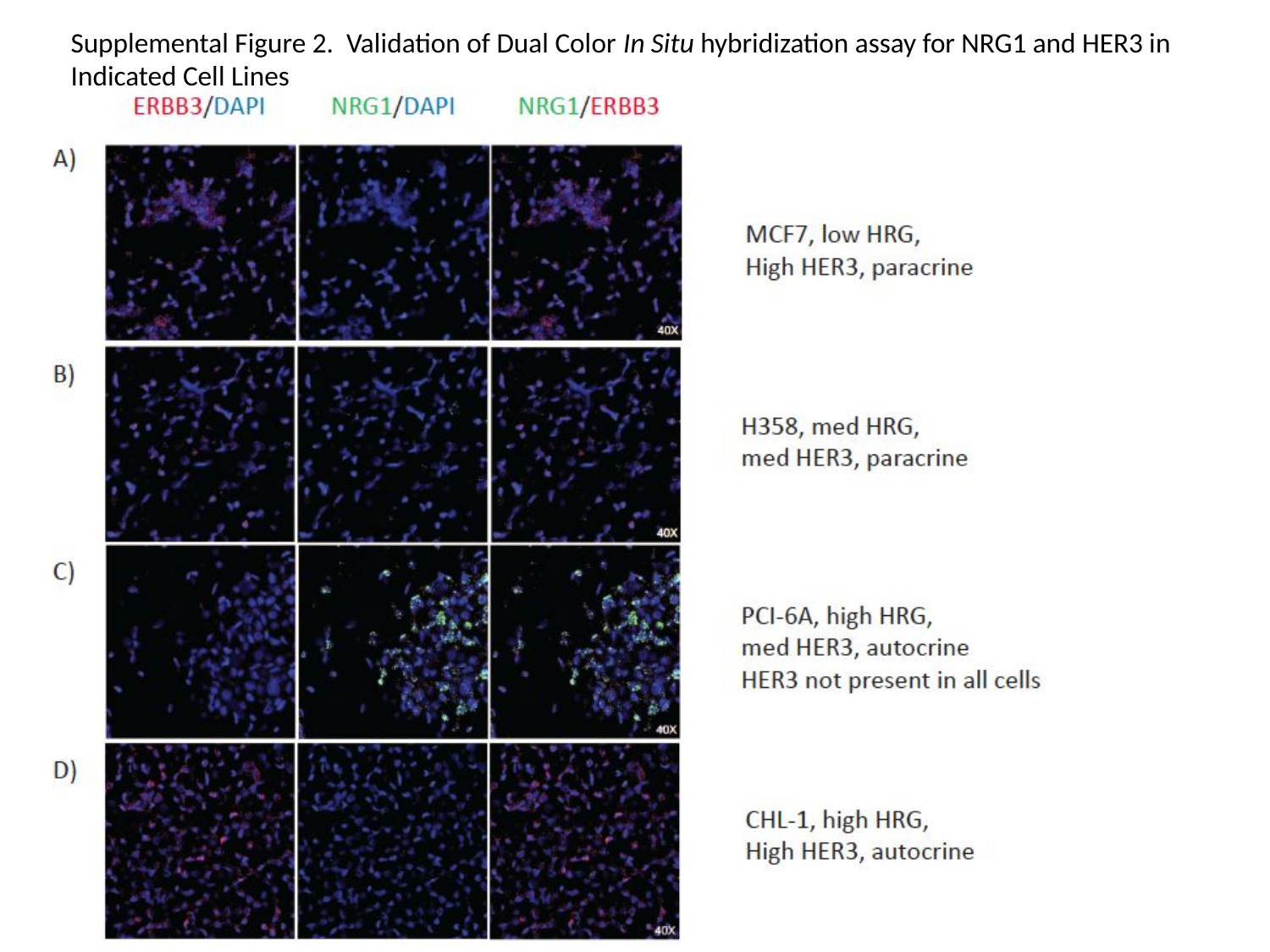

# Supplemental Figure 2. Validation of Dual Color In Situ hybridization assay for NRG1 and HER3 in Indicated Cell Lines
