# Supplementary figures and images for: High Heregulin Expression Is Associated with Activated HER3 and May Define an Actionable Biomarker in Patients with Squamous Cell Carcinomas of the Head and Neck
Source: PLoS One. 2013 Feb 28;8(2):e56765. doi: 10.1371/journal.pone.0056765 (PMC3586092; doi:10.1371/journal.pone.0056765)

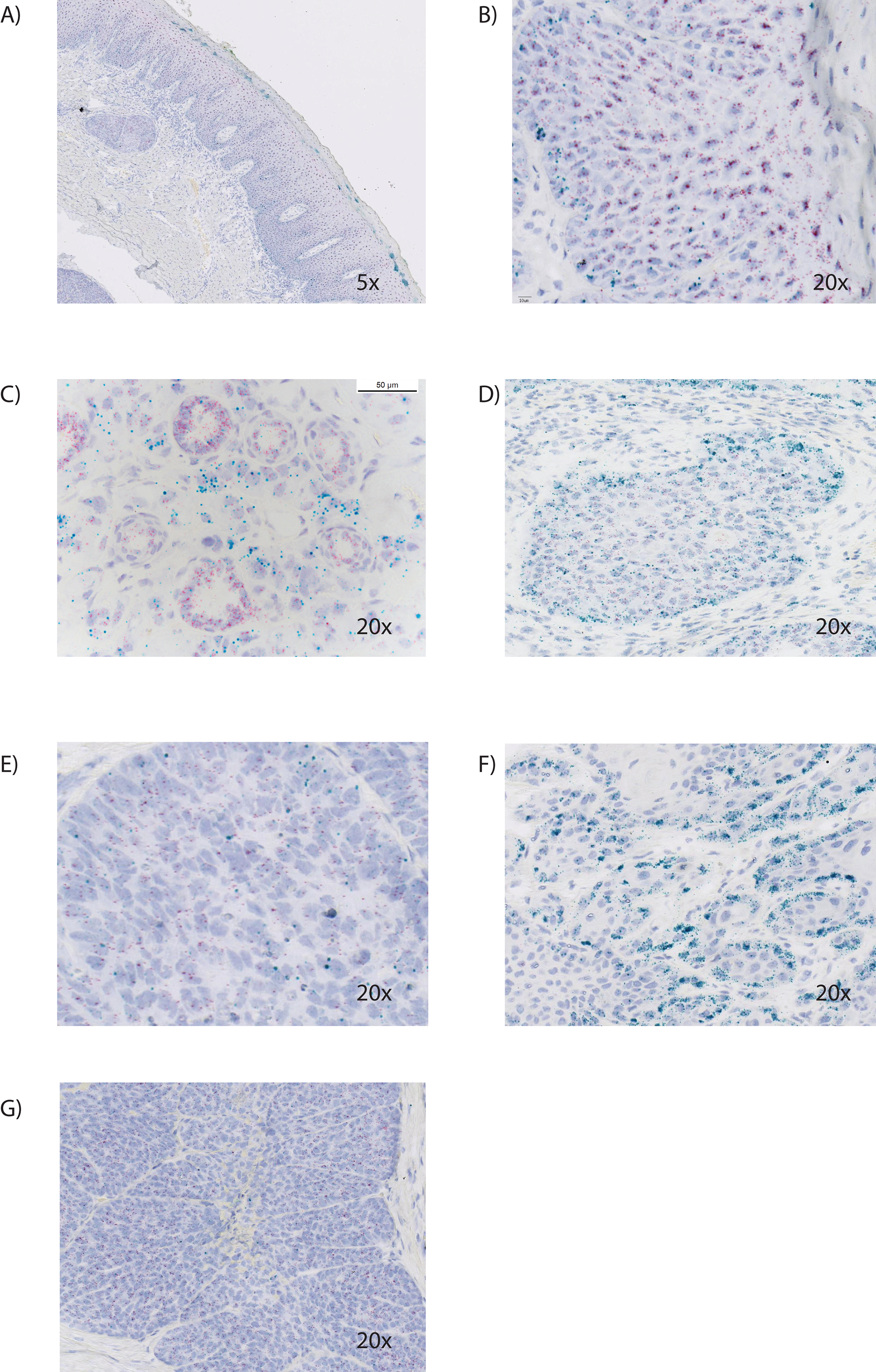

Supplement: Figure S3 — Additional examples of the colormetric dual color In Situ hybridization assay for HRG and HER3 in benign and malignant head and neck tissues. (TIF) [file pone.0056765.s003.tif]
